# A case of *Vagococcus fluvialis* isolated from the bile of a patient with calculous cholecystitis

**DOI:** 10.1186/s12879-023-08696-w

**Published:** 2023-10-16

**Authors:** Dan Zhang, Xiaosu Wang, Jingdan Yu, Zheng Dai, Qichao Li, Litao Zhang

**Affiliations:** Department of Clinical Laboratory, Wuhan Asia General Hospital, Wuhan Asia General Hospital, Wuhan University of Science and Technology, Wuhan, Hubei Province 430056 People’s Republic of China

**Keywords:** *Vagococcus fluvialis*, Chronic cholecystitis, Gallbladder stones, Bacterial identification

## Abstract

**Background:**

Chronic cholecystitis, characterized by persistent inflammation of the gallbladder, predominantly stems from the prolonged presence of gallstones. Calculous cholecystitis has demonstrated a consistent escalation in its incidence over time.Gallbladder stones have been recognized as a predisposing factor for the development of biliary tract infections.Concomitantly, there have been substantial shifts in the distribution and resistance profiles of pathogenic microorganisms responsible for biliary tract infections. The timely acquisition of bile samples for pathogen analysis is of paramount importance, given its critical role in guiding judicious clinical pharmacotherapy and enhancing patient prognosis.

**Case Presentation:**

We present a case involving a 66-year-old female patient who had previously undergone subtotal gastrectomy due to diffuse large B-cell lymphoma. The patient was admitted to our institution with complaints of abdominal pain. Subsequent diagnostic evaluation revealed concurrent choledocholithiasis and cholecystolithiasis. The patient underwent surgical cholecystectomy as the therapeutic approach. Histopathological examination of the excised gallbladder disclosed characteristic features indicative of chronic cholecystitis. Subsequent laboratory analysis of the patient’s bile specimen yielded Gram-positive cocci, subsequently identified through biochemical assays, mass spectrometry, and 16 S rRNA analysis as *Vagococcus fluvialis*. Further in vitro antimicrobial susceptibility testing using disk diffusion and microfluidic dilution showed that this strain exhibited inhibition zone diameters ranging from 12.0 to 32.0 mm in response to 26 antibiotics, including ampicillin, cefazolin, cefuroxime, cefotaxime, ceftriaxone, cefepime, ampicillin/sulbactam, piperacillin, ciprofloxacin, cefoperazone/sulbactam, imipenem, meropenem, piperacillin/tazobarb, penicillin, erythromycin, chloramphenicol, vancomycin, methotrexate/sulfamethoxazole, teicoplanin, linezolid, tigecycline, cefoxitin, ceftazidime, levofloxacin, minocycline and tobramycin. However, the inhibition zone diameters were 6.0 mm for amikacin, oxacillin, clindamycin, and tetracycline. The patient received ceftazidime anti-infective therapy both preoperatively and within 24 h postoperatively and was discharged successfully one week after surgery.

**Conclusion:**

In this study, we present the inaugural isolation and identification of *Vagococcus fluvialis* from bile specimens of patients afflicted with calculous cholecystitis. This novel finding lays a substantial experimental groundwork for guiding clinically rational antimicrobial therapy and advancing the exploration of relevant pathogenic mechanisms pertaining to *Vagococcus fluvialis* infections.

## Background

Chronic cholecystitis typically emerges from prolonged gallbladder inflammation attributed to the presence of gallstones or the recurrent migration of acute cholecystitis. Clinical manifestations exhibit considerable variability, ranging from asymptomatic or recurrent right upper quadrant discomfort to abdominal pain, and in some cases, acute exacerbations. A study by [1] reported a 44% positive bile culture rate in patients with chronic cholecystitis. Classification is based on the presence or absence of gallstones, distinguishing between calculous and acalculous cholecystitis. In recent years, shifting dietary habits, lifestyle changes, and widespread use of antimicrobial agents have contributed to an escalating incidence of calculous cholecystitis. Gallstone formation obstructs bile drainage, facilitating the entry of enteric pathogens into the biliary tract via blood, lymphatics, or retrograde flow, thereby instigating biliary tract infections (biliary tract infection,BTI). Subsequent infection further exacerbates disease progression, intensifying its severity and impeding effective clinical management [2].

*Vagococcus fluvialis* is a Gram-positive coccus that typically arranges singularly, in pairs, or in chains. It possesses flagella across its surface. It thrives within a temperature range of 10–40 °C and can tolerate growth in a 4% NaCl environment. However, it does not survive at 45 °C and in 6.5% NaCl. Additionally, exposure to 60 °C for 30 min is lethal for this organism [3]. As an opportunistic pathogen, *Vagococcus fluvialis* generally poses minimal threat to humans and other animals under standard conditions. Although predominantly isolated from animals or animal products [4–10], only rare instances of human infection have been reported [11–15]. Recently, our laboratory successfully isolated *Vagococcus fluvialis* from bile samples of patients with calculous cholecystitis. The isolated strain was subjected to biochemical assays, mass spectrometry identification, 16 S rRNA analysis, and antimicrobial susceptibility testing via disc diffusion and microbroth dilution methods. In view of the fact that there have been no reports of isolation of *Vagococcus fluvialis* from bile, the following is reported for the information of other laboratories.

## Case Presentation

The patient, a 66-year-old female, underwent subtotal gastrectomy five years ago for gastric lymphoma, histopathologically diagnosed as diffuse large B-cell lymphoma. She was recently admitted to our medical facility due to abdominal pain. On admission day, a comprehensive physical examination and pertinent investigations were conducted. The patient’s vital signs were recorded as follows: body temperature of 36.3 °C, heart rate of 76 beats per minute, respiratory rate of 19 breaths per minute, and blood pressure of 117/76 mmHg. Magnetic resonance imaging of the upper abdomen, pancreas, and biliary system revealed multiple findings: a cyst in the right hepatic lobe, multiple stones in the distal common bile duct, gallbladder stones, segmental discontinuity at the distal common bile duct, and slight pancreatic duct dilation.The patient’s stool appeared yellow-brown, and her laboratory parameters were as follows: total bilirubin of 7.8µmol/L, alanine aminotransferase of 22U/L, aspartate aminotransferase of 34U/L, alkaline phosphatase of 109U/L, gamma-glutamyl transferase of 14.2U/L, amylase of 108 U/L, and lipase of 27U/L. Peripheral blood analysis indicated a white blood cell count of 3.5 × 10^9/L, with neutrophils accounting for 46.2% of the total count.Admission diagnosis was (1) choledocholithiasis (2) gallbladder stones 2. chronic cholecystitis.

**Fig. 1:**
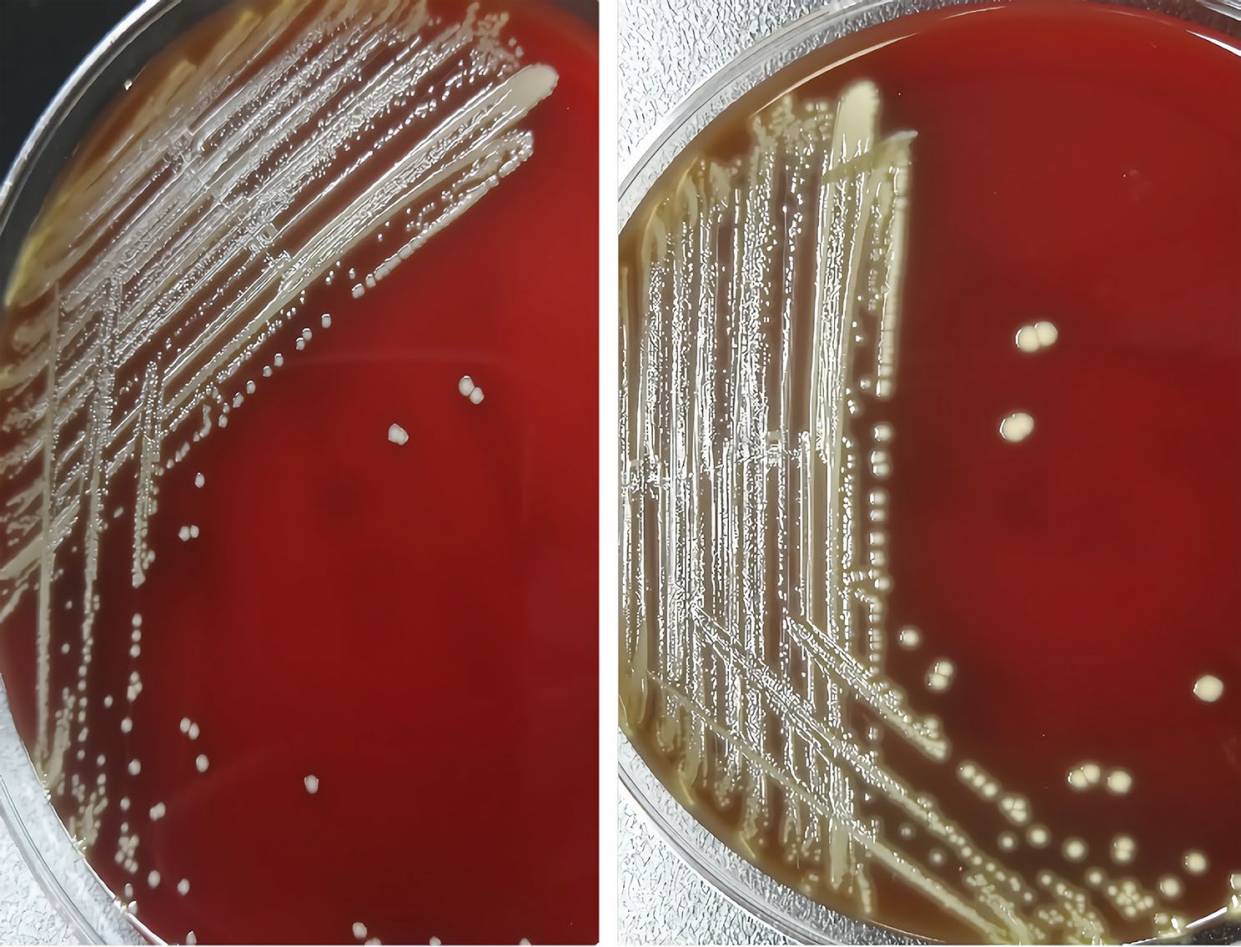
Morphology of blood agar plate colonies of *Vagococcus fluvialis* incubated at 35℃for 24 h (left) and 72 h (right)

**Fig. 2:**
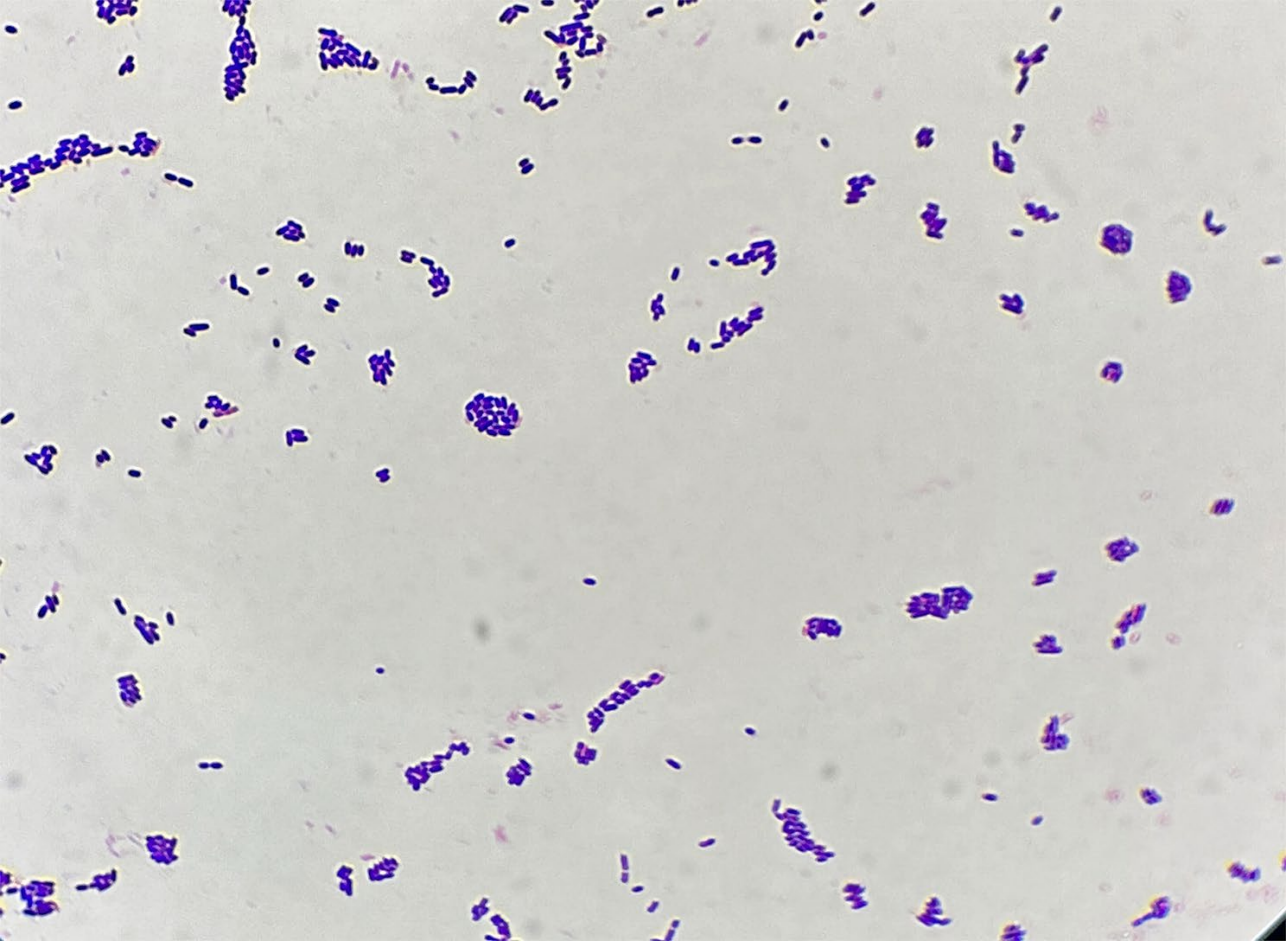
Gram staining of *Vagococcus fluvialis*

**Fig. 3:**
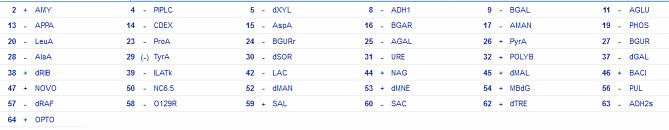
Biochemical reaction spectrum of *Vagococcus fluvialis*

**Fig. 4:**
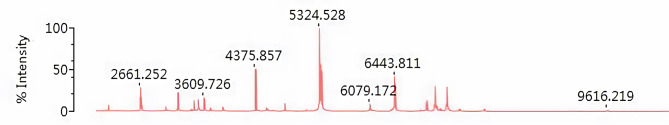
Mass spectrogram of *Vagococcus fluvialis*

Three days following admission, the patient underwent surgical cholecystectomy as treatment. Preoperative prophylactic antibiotic therapy with ceftazidime was administered. The excised gallbladder was sent for pathological examination, revealing evidence of chronic cholecystitis. On the evening of the surgery, the patient’s peripheral blood white cell count was recorded at 18.5 × 10^9/L. Continued ceftazidime therapy was implemented for infection. By the subsequent day, the peripheral blood white cell count had decreased to 12.2 × 10^9/L.During the surgical procedure, bile samples were collected and subsequently subjected to general bacterial culture in the laboratory. The bile samples were inoculated into blood culture bottles and Columbia blood agar plates. After 24 h of incubation(at 35℃), pinpoint-sized, circular, raised colonies were observed on the Columbia blood agar plates, exhibiting a neat and gray-white appearance, accompanied by slight alpha-hemolysis. Extending the incubation to 72 h, the colonies continued to grow, transitioning from gray-white to gray-yellow in color (Fig. 1). Microscopic examination of Gram staining revealed small, Gram-positive cocci arranged mostly in pairs or short chains, occasionally as individual bacterium (Fig. 2).Subsequent identification of the isolated microorganism was conducted using both the VITEK 2 compact automated bacterial identification system (bioMérieux) and matrix-assisted laser desorption/ionization time-of-flight mass spectrometry (MALDI-TOF MS, VITEK MS system, bioMérieux). These analyses yielded concordant results, identifying the organism as *Vagococcus fluvialis* with matching rates of 97.0% and 99.9% respectively. Biochemical reaction profiles and spectral peaks are illustrated in Figs. 3 and 4. Furthermore, 16 S rRNA sequencing analysis (The sequencing was conducted by Beijing Ruiboxingke Biotechnology Co., Ltd., using the Applied Biosystems sequencing platform, model 3730xl for DNA analysis.) displayed a remarkable 99.66% homology between the isolated strain and existing *Vagococcus fluvialis* sequences in the GenBank database(The sequencing report has been included in the supplementary material for the Editor’s reference).

Standardized drug sensitivity testing protocols for *Vagococcus fluvialis* remain elusive. In our study, we sought to assess drug sensitivity through both the disc diffusion method (using Mueller-Hinton agar, antimicrobial discs from Oxoid, UK) and microbroth dilution method(The bacterial sensitivity testing cards were purchased from bioMérieux). The results indicated that this *Vagococcus fluvialis* strain exhibited inhibitory zone diameters ≥ 20.0 mm for 21 antimicrobial agents including ampicillin, cefazolin, cefuroxime, cefotaxime, ceftriaxone, cefepime, ampicillin/sulbactam, piperacillin, ciprofloxacin, cefoperazone/sulbactam, imipenem, meropenem, piperacillin/tazobarb, penicillin, erythromycin, chloramphenicol, vancomycin, methotrexate/sulfamethoxazole, teicoplanin, linezolid, and tigecycline, with inhibitory zone diameters ranging from 7.0 to 19.0 mm for cefoxitin, ceftazidime, levofloxacin, minocycline, and tobramycin, and 6.0 mm for amikacin, oxacillin, clindamycin, and tetracycline. Microbroth dilution results demonstrated that 10 antimicrobial agents, including gentamicin, penicillin, erythromycin, vancomycin, methotrexate/sulfamethoxazole, teicoplanin, tigecycline, linezolid, daptomycin, and moxifloxacin, exhibited minimum inhibitory concentrations (MICs) of ≤ 1 µg/ml. Levofloxacin exhibited an MIC range of ≥ 2 µg/ml to < 4 µg/ml. Oxacillin, clindamycin, and rifampicin exhibited MICs of ≥ 4 µg/ml. For detailed results, refer to Table 1.One week post-surgery, the patient’s peripheral blood white cell count had normalized to 7.4 × 10^9/L. Liver and kidney function, as well as amylase levels, remained within normal parameters. The patient’s recovery progressed favorably, prompting her discharge on the same day, attesting to a successful postoperative outcome.

**Table 1:** Antimicrobial susceptibilities of *Vagococcus fluvialis*

Antimicrobial Agent	Disk Content	Zone diameter (mm)	MIC(µg/mL)
Ampicillin	10ug	28	/
Cefazolin	30ug	20	/
Cefoxitin	30ug	12	/
Cefuroxime	30ug	22	/
Ceftazidime	30ug	19	/
Cefotaxime	30ug	24	/
Cefatriaxone	30ug	26	/
Cefepime	30ug	27	/
Ampicillin /Sulbactam	1:1,20ug	29	/
Piperacillin	100ug	25	/
Ciprofloxacin	5ug	23	/
Levofloxacin	5ug	19	2
Gentamicin	10ug	/	1
Amikacin	30ug	6	/
Cefoperazone /Sulbactam	75:30,105ug	20	/
Imipenem	10ug	29	/
Meropenem	10ug	29	/
Piperacillin /Tazobactam	10:1,110ug	28	/
Minocycline	30ug	14	/
Tobramycin	10ug	15	/
Penicillin	10unit	28	0.5
Oxacillin	1ug	6	≥ 4
Erythromycin	15ug	31	≤ 0.25
Chloramphenicol	30ug	27	/
Clindamycin	2ug	6	≥ 4
Tetracycline	30ug	6	/
Vancomycin	30ug	22	≤ 0.5
Methotrexate/Sulfamethoxazole	19:1,25ug	28	≤ 0.5/10
Teicoplanin	30ug	21	≤ 0.5
Linezolid	30ug	32	1
Tigecycline	15ug	23	≤ 0.125
Daptomycin	/	/	0.25
Rifampicin	/	/	16
Moxifloxacin	/	/	1

Note: “/” was not tested or not applicable

## Discussion and conclusion

The prevailing literature on pathogenic bacterial isolates from bile predominantly features *Escherichia coli, Klebsiella pneumoniae, Enterococcus faecalis, Enterococcus faecium*, and *Pseudomonas aeruginosa* [15]. However, instances of pathogenicity attributable to *Vagococcus fluvialis* have yet to be documented. Antimicrobial susceptibility test for strains isolated from bile culture were performed in 557 hospitals in china [16].WHONET 5.6 software was used for statistical analysis.Results A total of 13,596 bacterial strains were isolated, inclu-ding gram-positive cocci 3698 strains, accounting for 27.2%, gram-negative bacilli 9898 strains, accounting for 72.8%.The top two gram-positive cocci are *Enterococcus faecalis* 1237 strains (9.1%) and *Entero-coccus faecium* 1198 strains (8.8%).The top three gram-negative ba-cilli are *Escherichia coli* 4311 strains ( 31.7%), *Klebsiella pneumoniae* 1518 strains ( 11.2%) and *Pseudomonas aeruginosa* 839 strains (6.2%). These findings underscore Gram-negative bacteria as the primary pathogenic agents in chronic cholecystitis. The authors postulate that widespread antimicrobial use has led to an upsurge in bacterial resistance and an increase in mixed infections, thereby altering the spectrum of infectious agents and presenting novel challenges in the management of chronic cholecystitis. These findings prompt a focused approach towards preventing infections from gut microbiota, the main causative agents in chronic cholecystitis.

*Vagococcus fluvialis*, a species within the *Vagococcus genus*, was initially reported by Collins et al. in 1989 [17]. Subsequent investigations demonstrated its presence in human blood, peritoneal fluid, wounds [18], oral cavity lesions [19], infectious endocarditis [20], and skin, tissue, and joint infections, particularly in patients with diabetes [14]. These studies suggest that, like other opportunistic pathogens, *Vagococcus fluvialis* has the potential to cause human infections under specific circumstances such as changes in colonization sites, compromised immunity, or microbial imbalances. In our study, *Vagococcus fluvialis* was isolated from the bile of a patient with calculous cholecystitis. This patient, who had undergone gastric lymphoma surgery, exhibited compromised immunity. Without timely surgical intervention, the risk of developing *Vagococcus fluvialis*-related biliary tract infection was imminent.

Currently, the identification methods for *Vagococcus fluvialis* primarily include biochemical identification, MALDI-TOF MS, and 16 S rRNA sequencing [14, 17, 20]. In this study, all three methods were employed to identify the isolated strain, each yielding highly confident results (97%, 99.9%, and 99.66%). This ensured the reliability of the identification outcomes. Considering operational convenience, the laboratory may preferentially opt for the biochemical identification method and MALDI-TOF MS to identify this strain.

Currently, reports on the drug sensitivity of *Vagococcus fluvialis* vary. Teixeira et al. [18] initially conducted MIC-based drug sensitivity testing for 25 antibiotics in 1997 and found that the four isolated strains were sensitive to ampicillin, cefotaxime, methotrexate/sulfamethoxazole and vancomycin, while being resistant to clindamycin, lomifioxacin and ofloxacin. In 2021, Racero L employed the Vitek™ 2 C automated system to conduct drug sensitivity testing and identified the best-performing antibiotics as ampicillin, methotrexate/sulfamethoxazole, vancomycin, teicoplanin, and linezolid [14]. In 2019, Chinese researchers [21] conducted Kirby-Bauer disk diffusion testing using Mueller-Hinton agar for *Vagococcus fluvialis* isolated from bone infections and found that linezolid, erythromycin, tetracycline, ampicillin, and chloramphenicol exhibited inhibition zones larger than 20.0 mm, while cefazolin, penicillin, levofloxacin, vancomycin, ciprofloxacin, gentamicin and ceftriaxone displayed inhibition zones ranging from 10.0 to 20.0 mm. Clindamycin, oxacillin and methotrexate/sulfamethoxazole displayed inhibition zones of 6.0 mm. Our laboratory’s results from both the disk diffusion method and the microdilution method showed that this bacterium was sensitive to most antibiotics, but resistant to amikacin, oxacillin, clindamycin, and tetracycline, in line with the aforementioned studies [14, 21, 22]. Notably, the results of antibiotic susceptibility testing obtained from both methods demonstrated good concordance. Seven antibiotics, including penicillin, erythromycin, vancomycin, linezolid, teicoplanin, and tigecycline, exhibited inhibition zones ≥ 20.0 mm and MICs ≤ 1 µg/ml; levofloxacin exhibited an inhibition zone of 19.0 mm and MIC of 2 µg/ml; while oxacillin and clindamycin exhibited inhibition zones of 6.0 mm and MICs ≥ 4 µg/ml. These findings indicate a strong correlation between the two methods of drug sensitivity testing. Based on operational feasibility, the laboratory may select either the disk diffusion method or the microdilution method for conducting drug sensitivity testing, depending on the specific circumstances.

Patients with chronic cholecystitis typically do not necessitate antibiotic treatment [22]. However, in cases of acute exacerbations, empirical antibiotic therapy can be considered, followed by tailored antibiotic selection based on pathogen identification and susceptibility testing [23]. In this case, the patient received prophylactic antimicrobial treatment with cefotetan for 24 h on the day of surgery. Following the surgery, and after two days when antibiotic susceptibility results became available, the patient did not receive any further antibiotic treatment. The patient recovered and was discharged one week postoperatively. Therefore, it is postulated that this *Vagococcus fluvialis* might be a normal resident of the biliary tract or a colonic organism that retrogradely entered the biliary tract.

In essence, this study marks the pioneering isolation of *Vagococcus fluvialis* from bile samples of individuals with calculous cholecystitis. The identification process involved three distinct methods: biochemical reactions, mass spectrometry, and 16 S rRNA analysis. Furthermore, the bacterial strain’s antimicrobial susceptibility was assessed through both the Kirby-Bauer disk diffusion method and the minimum inhibitory concentration (MIC) approach. These findings hold crucial clinical significance, serving as a guide for rational antibiotic usage and enhancing patient outcomes. Simultaneously, they lay a solid experimental foundation for guiding judicious antibiotic strategies and investigating the underlying pathogenic mechanisms associated with *Vagococcus fluvialis* infections.

## Data Availability

The results of 16 S rRNA sequencing (Beijing Ruibo Xingke Biotechnology Co., Ltd.) analysis in this study have been provided in the Supplementary Material and are available for editorial review.
